# Association of acute blood biomarkers with diffusion tensor imaging and outcome in patients with traumatic brain injury presenting with GCS of 13–15^☆^

**DOI:** 10.1016/j.nicl.2025.103934

**Published:** 2025-12-18

**Authors:** Malla Mononen, Mehrbod Mohammadian, Iftakher Hossain, Timo Roine, Olli Tenovuo, Kaj Blennow, Jessica Gill, Mark van Gils, Peter Hutchinson, Teemu M. Luoto, Henna-Riikka Maanpää, David K. Menon, Virginia F.J. Newcombe, Rahul Raj, Jean-Charles Sanchez, Riikka S.K. Takala, Jussi Tallus, Henrik Zetterberg, Jussi P. Posti

**Affiliations:** a Neurocenter, Department of Neurosurgery, Turku University Hospital, Finland; b Turku Brain Injury Center, Turku University Hospital, Finland; c Department of Clinical Neurosciences, University of Turku, Finland; d Athinoula A. Martinos Center for Biomedical Imaging, Department of Radiology, Massachusetts General Hospital, Harvard Medical School, Boston, MA, USA; e Department of Clinical Neurosciences, Neurosurgery Unit, University of Cambridge, Addenbrooke’s Hospital, Cambridge, the United Kingdom of Great Britain and Northern Ireland; f Turku Brain and Mind Center, University of Turku, Turku, Finland; g Department of Neuroscience and Biomedical Engineering, Aalto University School of Science, Espoo, Finland; h Institute of Neuroscience and Physiology, Department of Psychiatry and Neurochemistry, The Sahlgrenska Academy at the University of Gothenburg, Mölndal, Sweden; i Clinical Neurochemistry Laboratory, Sahlgrenska University Hospital, Mölndal, Sweden; j National Institute of Nursing Research, National Institutes of Health, Bethesda, MD, USA; k Faculty of Medicine and Health Technology, Tampere University, Tampere, Finland; l Department of Neurosurgery, Tampere University Hospital and Tampere University, Tampere, Finland; m Division of Anaesthesia, University of Cambridge, Addenbrooke’s Hospital, Cambridge, the United Kingdom of Great Britain and Northern Ireland; n Department of Neurosurgery, Helsinki University Hospital and University of Helsinki, Helsinki, Finland; o Department of Specialities of Internal Medicine, Faculty of Medicine, University of Geneva, Geneva, Switzerland; p Perioperative Services, Intensive Care Medicine and Pain Management, Turku University Hospital and University of Turku, Finland; q Department of Radiology, University of Turku and Turku University Hospital, Turku, Finland; r Department of Molecular Neuroscience, UCL Institute of Neurology, Queen Square, London, the United Kingdom of Great Britain and Northern Ireland; s UK Dementia Research Institute at UCL, University College London, London, the United Kingdom of Great Britain and Northern Ireland; t Hong Kong Center for Neurodegenerative Diseases, Hong Kong, China; u Wisconsin Alzheimer’s Disease Research Center, University of Wisconsin School of Medicine and Public Health, University of Wisconsin-Madison, Madison, WI, USA

**Keywords:** Blood-based biomarker, Traumatic brain injury, Outcome, Diffuse axonal injury, Diffusion tensor imaging

## Abstract

The aim of the study was to assess the association between blood-based biomarkers of different cellular origins and later white matter integrity, measured using post-acute diffusion tensor metrics, and their relation to outcome in patients presenting with Glasgow Coma Scale of 13–15 after traumatic brain injury (TBI).

Admission plasma samples for glial fibrillary acidic protein (GFAP), interleukin 10 (IL-10), heart fatty-acid binding protein (H-FABP), S100 calcium-binding protein B (S100B), total tau (T-tau), and amyloid beta 40 and 42 (Aβ40 and Aβ42) were taken for 92 patients. Diffusion-weighted magnetic resonance imaging (DW-MRI) and outcome evaluation was done ≥ 90 days post-injury. Outcome was assessed using Glasgow Outcome Scale-Extended (GOSE) and dichotomized as complete (GOSE 8) and incomplete (GOSE < 8) recovery. Mean fractional anisotropy (FA), mean diffusivity (MD), axial diffusivity (AD), and radial diffusivity (RD) were calculated from the skeletonized white matter tracts of the whole brain.

IL-10 and T-tau showed significant weak-moderate negative correlations with FA, and significant positive correlations with MD and RD in incompletely recovered patients. GFAP showed significant weak positive correlations with MD and RD, while its correlation with FA was slightly below significance threshold after correction for multiple comparison in incompletely recovered patients. Similar trends were observed in the whole cohort and in the CT-positive cohort, although these did not reach statistical significance.

Higher acute levels of GFAP, IL-10 and T-tau may be associated with the development of axonal injury. If validated in future studies, these biomarkers may help identify patients who require closer follow-up and DW-MRI.

## Highlights

• 92 mild TBI patients with biomarkers at admission and DTI ≥ 90 days post-injury.
• Reduced white matter integrity associates with biomarkers in incomplete recovery.
• Fractional anisotropy associates with interleukin 10 (IL-10) and tau.
• Mean and radial diffusivity relate to IL-10, tau and glial fibrillary acidic protein.
• Similar but non-significant trends were observed in the full and CT-positive cohorts.

## Graphical abstract

**Figure:**
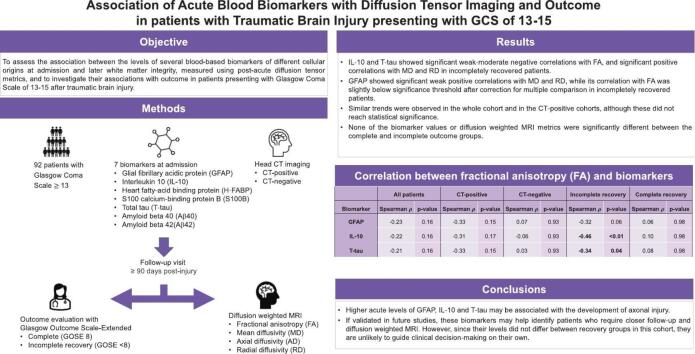

## 1 Introduction

Traumatic brain injury (TBI) represents a significant burden to public health care worldwide with over 50 million reported new cases annually. (Maas et al., 2022) Mild TBI (mTBI) accounts for approximately 90 % of the total number TBI cases. (Steyerberg et al., 2019) Many patients with mTBI fully recover within three months after the initial injury, but a significant percentage develops prolonged or persistent post-injury symptoms such as fatigue, cognitive problems, and depression. (Nelson et al., 2019, Mikolić et al., 2021, Cancelliere et al., 2023).

Conventional imaging modalities such as computed tomography (CT) and standard magnetic resonance imaging (MRI), have poor sensitivity in detecting microscopic axonal damage in brain white matter (WM) following TBI, which is known as diffuse axonal injury (DAI). (Shenton et al., 2012, Hutchinson et al., 2018, Benjamini et al., 2021) Pathophysiological changes associated with DAI are identified as one of the main causes for incomplete recovery in mTBI, (Niogi et al., 2008, Johnson et al., 2013) as suggested by human neuropathological studies. (Bigler, 2004, Blumbergs et al., 1994) DAI can potentially be detected using advanced diffusion-weighted (DW) MRI methods, such as diffusion tensor imaging (DTI), where the movement of water in WM is measured. (Basser et al., 1994) Alterations in WM integrity and axonal directionality are expressed as reduced fractional anisotropy (FA) and increased diffusivity measures in the acute (Gu et al., 2013) and chronic (Kraus et al., 2007, Perez et al., 2014) phases of TBI. FA and mean diffusivity (MD) are thought to represent the overall degree of WM integrity; elevated radial diffusivity (RD) has been linked with demyelination, while axial diffusivity (AD) reflects more direct axonal damage. (Winklewski et al., 2018, Metting et al., 2007) Disruptions in WM integrity, characterized by these aforementioned DTI metrics, have also been reported to relate to outcome. (Metting et al., 2007, Sidaros et al., 2008) DTI metrics from DW-MRI can be quantified with a global approach of entire brain or with particular areas of focus. In this study, we used the global approach.

An association between the axonal biomarker neurofilament light (NfL) and DTI metrics in mTBI has been reported. (Hossain et al., 2023, Ljungqvist et al., 2017, Shahim et al., 2020, Newcombe et al., 2022) Amyloid-β biomarkers (Aβ40 and Aβ42) are well-studied neurodegenerative markers (Blennow et al., 2006, Khan and Bloom, 2016), but no significant relationship to WM injury after a TBI has been found. (Lippa et al., 2019) Total tau (T-tau) has shown associations with DTI metrics in a longitudinal study of sports-related concussions. (Wu et al., 2023).

Astroglial cells release S100 calcium-binding protein B (S100B) and glial fibrillary acidic protein (GFAP) after TBI, and GFAP has been shown to associate with WM integrity (Shahim et al., 2020, Newcombe et al., 2022, Yue et al., 2019) in patients with mTBI. Admission S100B predicts traumatic findings in head CT after mTBI (Undén et al., 2013), but has not been reported to be associated with axonal injury findings on DW-MRI (Metting et al., 2012). Neuroinflammation marker interleukin 10 (IL-10) and brain injury marker heart fatty-acid binding protein (H-FABP) have gained interest in the acute diagnostics and outcome prediction (Lagerstedt et al., 2020, Mahatma et al., 2012), but no studies have explored their association with WM integrity changes after TBI.

Because DAI evolves over weeks to months, an association between admission biomarker levels and later DTI findings would be consistent with these biomarkers reflecting early processes that lead to DAI, rather than only acute effects. (de Macruz, et al., 2022) Documenting the presence of DAI at a fairly early phase after the injury has clinical implications for outcome prediction, the need for follow-up and care, medicolegal aspects, and will be a prerequisite for eventual targeted therapies. Performing DTI in all symptomatic patients with mTBI is neither cost-effective nor logistically feasible. A readily available objective screening tool such as peripheral blood biomarkers would facilitate the selection of patients for DTI. Preliminary evidence shows that GFAP and NfL have promise in this setting. (Richter et al., 2024) We conducted an association study between acute biomarker levels in various cell types and white matter integrity, as measured by diffusion tensor metrics. We also investigated their relations with outcome in patients with mTBI.

## 2 Methods

### 2.1 Study population

**Fig. 1:**
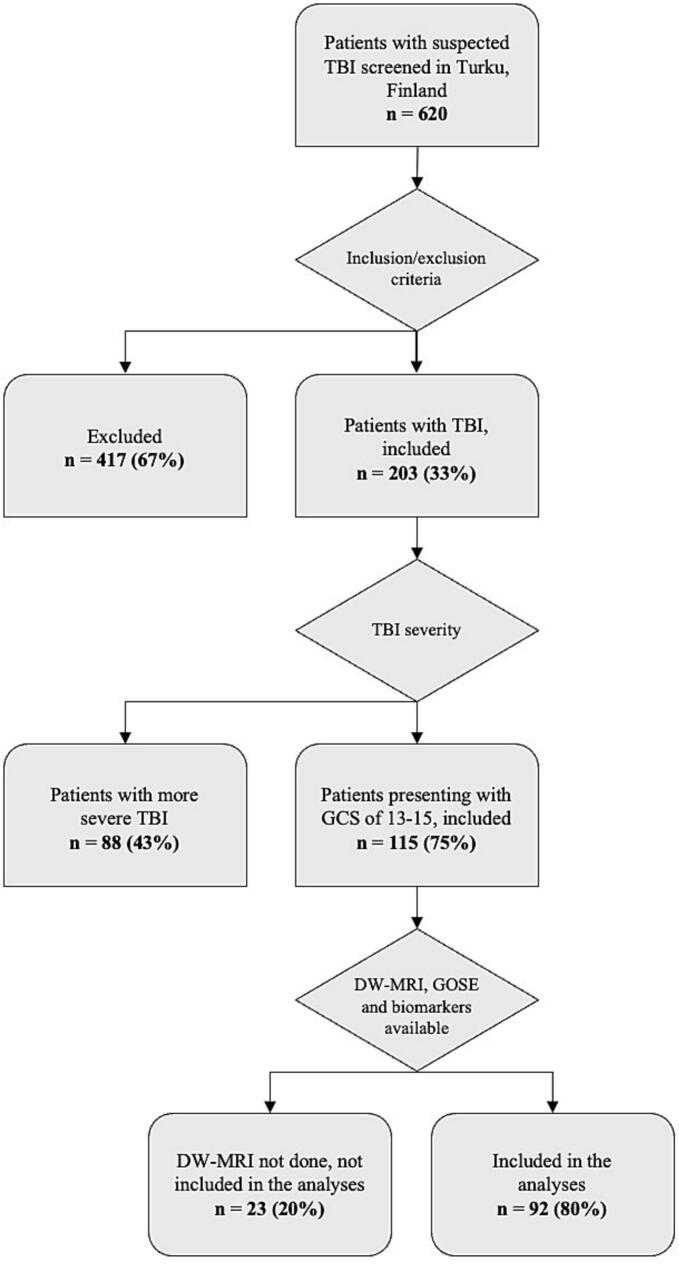
Study recruitment flow chart. TBI, traumatic brain injury; GCS, Glasgow Coma Scale; DW-MRI, diffusion-weighted magnetic resonance imaging; GOSE, Glasgow Outcome Scale Extended.

The study recruited patients with TBI presenting with Glasgow Coma Scale (GCS) score of 13–15 in Turku University Hospital between November 2011 and October 2013 and was a part of the EU-supported TBIcare-project (Evidence-based Diagnostic and Treatment Planning Solution for Traumatic Brain Injuries). Blood biomarkers were sampled upon arrival to the hospital within a 24 h timeframe. For the purposes of this study, only those who underwent late DW-MRI ≥ 90 days post-injury were included, resulting with a total number of 92 patients, as illustrated in a flow chart in Fig. 1.

Patients were included in the study based on previously described TBI criteria (Kay et al., 1993), age ≥ 18 years, worst recorded Glasgow Coma Scale (GCS) score ≥ 13, and presenting with a need for a urgent head CT according to the NICE criteria (National Clinical Guideline Centre (UK), 2014).

Patients were excluded if any of the following criteria were met: a head CT was not taken during admission, age < 18 years, chronic subdural hematoma, blast-induced or penetrating injury, more than two weeks from the injury, pre-existing neurological disorder preventing individual from living independently, non-Finnish speaker, unable to attend follow-up visits due to remote residence or consent not obtained.

The study protocol was accepted by the ethical review board of the Hospital District of Southwest Finland (68/180/2011). Patients or their family members were provided information both verbally and in writing, and a written consent was collected from all participants.

### 2.2 TBI severity and outcome

The severity of TBI was based only on the worst GCS score recorded, and it was examined by paramedics at the accident site or in the ambulance, or during admission to hospital by an emergency physician. Three experienced neurosurgeons and neurotrauma researchers (J.P.P, T.L, R.R) examined the head CT scans using the Marshall classification. (Marshall et al., 1992) Head CT scans classified as Marshall I were regarded as CT-negative and Marshall II-VI injuries were included in the CT-positive cohort. To better characterize the patient population, overall injury severity was presented using the Injury Severity Score (ISS). (Linn, 1995).

Outcome assessment was done during follow-up visit ≥ 90 days post-injury (median = 231, IQR = 195–264 days) with the Glasgow Outcome Scale-Extended (GOSE). (Wilson et al., 1998) GOSE is a structured interview to assess functional outcome on an eight-point scale and is often used as a primary outcome measure in TBI studies. Evaluation was done in close proximity (mean = 1.2 days, standard deviation (SD) = 9.3 days) to the DW-MRI scan. The results were dichotomized to complete (GOSE 8) or incomplete recovery (GOSE < 8) to reflect mild TBI recovery. The length of post-traumatic amnesia (PTA) was assessed using the Rivermead method, a retrospective questioning protocol used to determine when the patient regained continuous memory after TBI. (King et al., 1997) The evaluation was performed by a senior neurologist (O.T.), with DW-MRI and biomarker results blinded.

### 2.3 Biomarker analyses

Biomarker samples were collected from peripheral blood soon after the admission to the ED. Time elapsed from injury to sampling was categorized into samples collected within 24 h and after 24 h of injury. Samples were drawn into EDTA-tubes and quickly placed on ice. Further separation of the plasma was done within 1 h and the samples were preserved at –80 °C until the analyses. In rare case of hemolyzed samples, they were excluded from further analyses. Laboratory technicians performing the biomarker analyses were board-certified and blinded to clinical data.

The GFAP and T-tau plasma concentrations were measured with the Human Neurology 4-Plex A assay on an HD-1 Single molecule array (Simoa) in Mölndal, Sweden according to the standardization from the manufacturer (Quanterix, Billerica, MA, USA). For GFAP, the lower limit of detection for (LLoD) was 0.22 pg/mL. The lower limit of quantification (LLoQ) for GFAP was 0.47 pg/mL and the calibration range was 0.99 pg/mL to 725.0 pg/mL. For T-tau, LLoD was 0.02 pg/mL, LLoQ was 0.05 pg/mL, with a calibration range between 0.14 pg/mL and 112.0 pg/mL.

H-FABP and IL-10 plasma samples were measured using the K151HTD and K151QUD kits, respectively, from Meso Scale (Meso Scale Diagnostics, Rockville, MD, USA). For H-FABP, LLoD was 0.10 ng/mL, and the calibration range was 0.14–100 ng/mL. The test for H-FABP lacks full validation by Meso Scale and has no LLoQ. For IL-10, LLoD was 0.04 pg/mL, LLoQ was 0.30 pg/mL, with the calibration range of 0.08–317.0 pg/mL.

S100B levels were analyzed using EZHS100B-33 K kit from Millipore (Millipore, Billerica, MA, USA) according to the manufacturer’s instructions. H-FABP, IL-10, and S100B analyses were conducted in Geneva, Switzerland. LLoD for S100B was 2.7 pg/mL and the calibration range was from 2.7 to 2000.0 pg/mL. A single patient had levels below the detection range, and we assigned a concentration level of 1 pg/mL to this patient, with no impact to the statistics obtained.

Aβ40 and Aβ42 plasma levels were analyzed using a duplex Simoa immunoassay (Quanterix, Billerica, MA, USA) in Bethesda, MD, USA. Aβ40 had LLoD of 0.05 pg/mL, LLoQ of 0.14 pg/mL, and calibration ranged from 0 pg/mL to 90.0 pg/mL. For Aβ42 the concentration of LLoD was 0.14 pg/mL, LLoQ was 0.69 pg/mL, and the calibration range was 0––11.0 pg/mL.

### 2.4 MRI acquisition

The MRIs were done using a Siemens Verio 3 T scanner at Turku University Hospital. Axial DW-MR images were acquired using a spin-echo echo-planar imaging sequence with a field of view of 192 × 192 mm, echo time of 106 ms, and repetition time of 11.7 sec, resulting in images with a 2 × 2 × 2 mm voxel size and 77 axial slices. DW-MRI data were acquired with a b-value of 1000 sec/mm^2^ in 64 gradient directions distributed uniformly on a unit sphere. (Jones et al., 1999) Additionally, susceptibility-weighted imaging, fluid-attenuated inversion recovery, T2-weighted, and structural T1-weighted images were obtained from each subject. Post-acute WM integrity was described as fractional anisotropy (FA) and mean diffusivity (MD), as well as axial (AD) and radial diffusivity (RD).

### 2.5 DTI processing and analyses

Subject motion and eddy current distortion artifacts in the DW-MR images were adjusted utilizing ExploreDTI. (Leemans and Jones, 2009) Images were further analyzed using tract-based spatial statistics (Kay et al., 1993), a whole-brain voxel-wise method in the FMRIB Software Library (Smith et al., 2006). FMRIB_FA template was used to reconstruct a WM FA skeleton into which the individual voxel-wise FA values were projected. Similar steps were applied to non-FA DTI measures, where MD, AD, and RD levels were projected into the whole brain skeletonized WM tracts. Average values from the whole WM tract skeleton were calculated for all of the DTI metrics.

### 2.6 Statistical analysis

Data normality was evaluated using the Shapiro-Wilk test and by visually inspecting the histograms. All numeric variables, including age, ISS, biomarker levels, and DTI metrics were not normally distributed, so the non-parametric Mann-Whitney *U* test was used to compare their group differences. Categorical variables were examined with the Chi-square test. Worst post-resuscitation GCS, cause of injury, Marshall grade, and GOSE were labeled as ordinal variables. The relations between DTI metrics and biomarkers in different patient groups were assessed with the partial Spearman rank correlation, using age and sex as covariates. To control for multiple biomarkers and DTI metrics in correlation analyses, the Benjamini-Hochberg correction was applied within each group of analyses. Statistical significance was defined as p-values < 0.05. Statistical analyses were conducted using R Statistical software (version 1.3.959) and IBM SPSS (Version 27).

## 3 Results

A total of 92 patients with TBI presenting with GCS of 13–15 were included, as shown in Fig. 1. Descriptive statistics for clinical and demographic characteristics by group (CT-positive, CT-negative, incomplete recovery, complete recovery) are presented in Table 1. There were 39 patients (42 %) with CT-positive findings, and 53 (58 %) patients had CT-negative scans. Further, 35 patients (38 %) had complete recovery (GOSE 8), and 57 (62 %) had incomplete recovery (GOSE < 8). Separate analyses were performed in CT-negative patients with complete (n = 24, 45 %) or incomplete (n = 29, 55 %) recovery. The exact time of injury was available for 74 % of the patients (n = 68). The median time elapsed from injury to blood sampling was 14.5 h (IQR = 6–30 h). Of the patients whose precise injury time was unknown, 8 patients had blood samples within 24 h, and 16 patients after 24 h from the injury. The median time from injury to DW-MR imaging was 231 days (IQR = 195–264 days, full range = 126–429 days). The median ISS was 5.5 (IQR = 1–12). Higher ISS values were observed in CT-positive group (p < 0.001) compared with the CT-negative groups, but the difference was not significant between outcome groups.

**Table 1:** Patient demographics and clinical characteristics in whole cohort and subgroups.

**Characteristic**	**All patients**	**CT-positive**	**CT-negative**	**p-value**	**Incomplete recovery**	**Complete recovery**	**p-value**
Number of patients (%)	92	39 (42.3)	53 (57.6)		57 (61.9)	35 (38.0)	
Age, median (IQR), y	47 (26–62)	51 (27.5–67)	46 (26–59)	0.148^a^	47 (30–60)	47 (22–64.5)	0.672^a^
Sex, No. (%)				0.051^b^			0.161^b^
Male	63 (68.4)	31 (79.4)	32 (60.3)		36 (63.1)	27 (77.1)	
Female	29 (31.5)	8 (20.5)	21 (39.6)		21 (36.8)	8 (22.8)	
Worst GCS, No. (%)				0.167^c^			0.676^c^
15	62 (67.3)	22 (56.4)	40 (75.4)		40 (70.1)	22 (62.8)	
14	24 (26.0)	14 (35.8)	10 (18.8)		13 (22.8)	11 (31.4)	
13	6 (6.5)	3 (7.6)	3 (5.6)		4 (7.0)	2 (5.7)	
Cause of injury, No. (%)				0.352^c^			0.617^c^
1 Road traffic crash	28 (30.4)	10 (25.6)	18 (33.9)		20 (35.0)	8 (22.8)	
2 Incidental fall	49 (53.2)	25 (64.1)	24 (45.2)		29 (50.8)	20 (57.1)	
3 Violence/assault	8 (8.6)	2 (5.1)	6 (11.3)		4 (7.0)	4 (11.4)	
4 Other non-intentional injury	7 (7.6)	2 (5.1)	5 (9.4)		4 (7.0)	3 (8.5)	
Isolated TBI, No. (%)	50 (54.3)	18 (46.1)	32 (60.3)	0.175^b^	28 (49.1)	22 (62.8)	0.199^b^
Extracranial injuries with TBI, No. (%)	42 (45.6)	21 (53.8)	21 (39.6)		29 (50.8)	13 (37.1)	
ISS, median (IQR)	5.5 (1–12)	9 (4–12)	2 (1–9)	**<0.001**^a^	8 (3–12)	4 (1–9)	0.066^a^
Admitted to hospital, No. (%)	67 (72.8)	35 (89.7)	32 (60.3)	**0.001**^b^	43 (75.4)	24 (68.5)	0.472^b^
Discharged from the ED, No. (%)	25 (27.1)	4 (10.2)	21 (39.6)		14 (24.5)	11 (31.4)	
CT-positive, No. (%)	39 (42.3)	39	0	**<0.001**^b^	28 (49.1)	11 (31.4)	0.095^b^
CT-negative, No. (%)	53 (57.6)	0	53		29 (50.8)	24 (68.5)	
Marshall grade, No. (%)				**<0.001**^c^			0.421^c^
Diffuse injury I, no visual pathology	53 (57.6)	0	53 (100)		29 (50.8)	24 (68.5)	
Diffuse injury II	25 (27.1)	25 (64.1)	0		17 (29.8)	8 (22.8)	
Diffuse injury III	3 (3.2)	3 (7.6)	0		3 (5.2)	0	
Diffuse injury IV	2 (2.1)	2 (5.1)	0		1 (1.7)	1 (2.8)	
Evacuated mass lesion	6 (6.5)	6 (15.3)	0		4 (7.0)	2 (5.7)	
Non-evacuated mass lesion	3 (3.2)	3 (7.6)	0		3 (5.2)	0	
Recovery, No. (%)				0.095^b^			**<0.001**^b^
Incomplete	57 (61.9)	28 (71.7)	29 (54.7)		57 (100)	0	
Complete	35 (38.0)	11 (28.2)	24 (45.2)		0	35 (100)	
GOSE, No. (%)				0.082^c^			**<0.001**^c^
3	4 (4.3)	4 (10.2)	0		4 (7.0)	0	
4	5 (5.4)	3 (7.6)	2 (3.7)		5 (8.7)	0	
5	3 (3.2)	1 (2.5)	2 (3.7)		3 (5.2)	0	
6	13 (14.1)	4 (10.2)	9 (16.9)		13 (22.8)	0	
7	32 (34.7)	16 (41.0)	16 (30.1)		32 (56.1)	0	
8	35 (38.0)	11 (28.2)	24 (45.2)		0	35 (100)	

Statistically significant p-values are in bold. SD, standard deviation; CT, computed tomographic; ISS, injury severity score; IQR, interquartile range; ED, emergency department; GOSE, Glasgow Outcome Scale-Extended. GOSE 1–7 = incomplete recovery, GOSE 8 = complete recovery.

a Mann–Whitney *U* test.

b Chi-square test.

c Fisher’s exact test.

### 3.1 Biomarker levels

The median biomarker levels in subgroups are shown in Supplementary **table S1**. The admission levels of GFAP, IL-10, H-FABP, and T-tau were significantly higher in CT-positive patients compared to CT-negative patients (p < 0.001, p = 0.011, p = 0.020, and p < 0.001, respectively, Fig. 2A). The levels of S100B, Aβ42, and Aβ40 were not significantly different between CT-positive and CT-negative patients (Fig. 2B). None of the biomarkers taken at the time of admission were significantly different between patients with incomplete recovery compared with patients with complete recovery, as shown in Fig. 2A, Fig. 2B, Fig. 3, Fig. 4.

**Fig. 2A:**
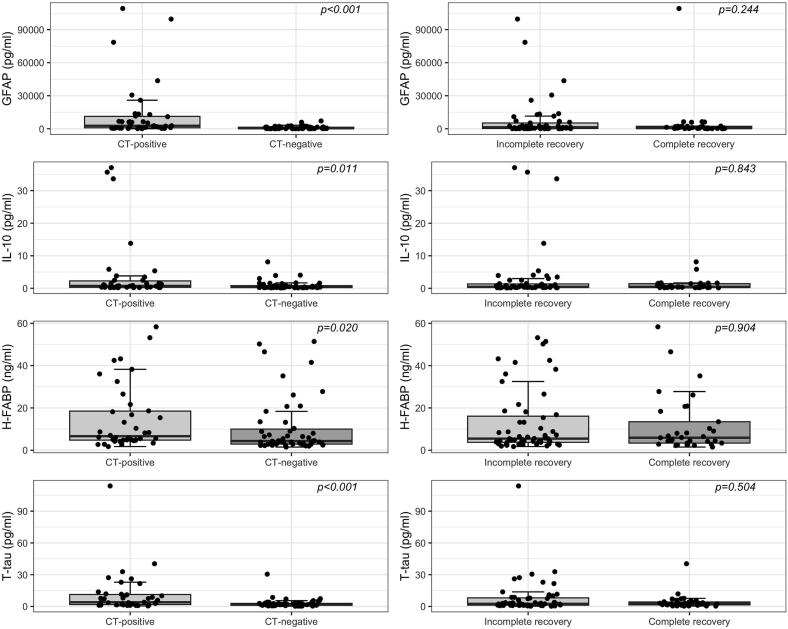
Comparison of biomarker levels of GFAP, IL-10, H-FABP, and T-tau in traumatic brain injury with abnormal cranial computed tomography (CT-positive) vs normal cranial CT (CT-negative) and incomplete recovery (GOSE < 8) vs complete recovery (GOSE = 8). Statistical significance was based on the Mann-Whitney test comparison. GFAP, glial fibrillary acidic protein; IL-10, interleukin 10; H-FABP, heart fatty-acid binding protein; T-tau, total tau; GOSE, Glasgow Outcome Scale Extended. One high outlier in T-Tau removed from visual presentation.

**Fig. 2B:**
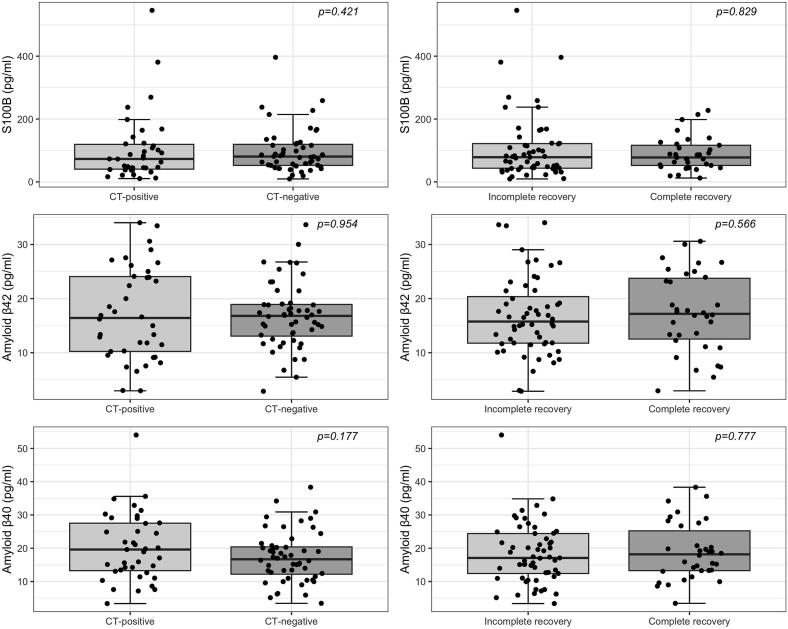
Comparison of biomarker levels of S100B, Aβ42, and Aβ40 in traumatic brain injury with abnormal cranial computed tomography (CT-positive) vs normal cranial CT (CT-negative) and incomplete recovery (GOSE < 8) vs complete recovery (GOSE = 8). Statistical significance was based on the Mann-Whitney test comparison. S100B, S100 calcium binding protein; Aβ42/ Aβ40, amyloid beta 42/40; GOSE, Glasgow Outcome Scale Extended.

**Fig. 3:**
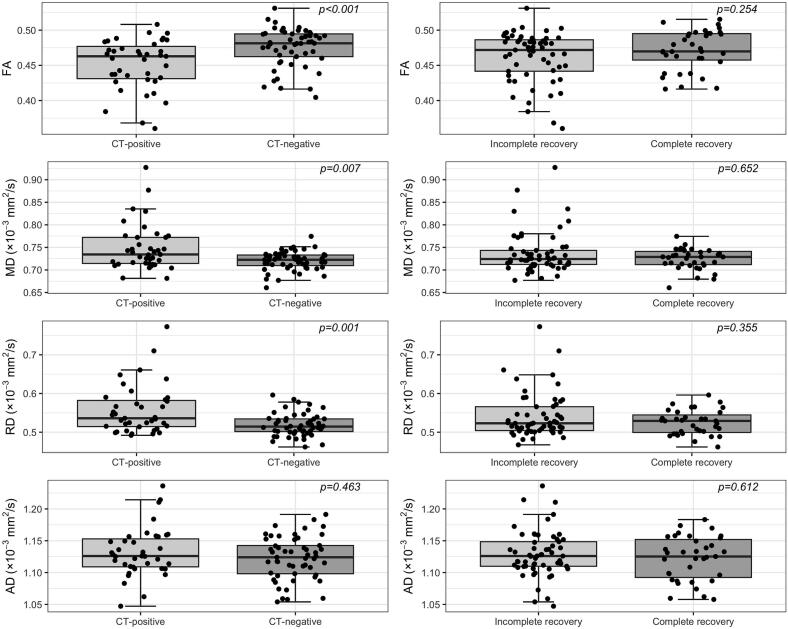
Comparison of DTI metrics FA, MD, RD, and AD in traumatic brain injury with abnormal cranial computed tomography (CT-positive) vs normal cranial CT (CT-negative) and incomplete recovery (GOSE < 8) vs complete recovery (GOSE = 8). Statistical significance was based on the Mann-Whitney test comparison. FA, fractional anisotropy; MD, mean diffusivity; RD, radial diffusivity; AD, axial diffusivity.

**Fig. 4:**
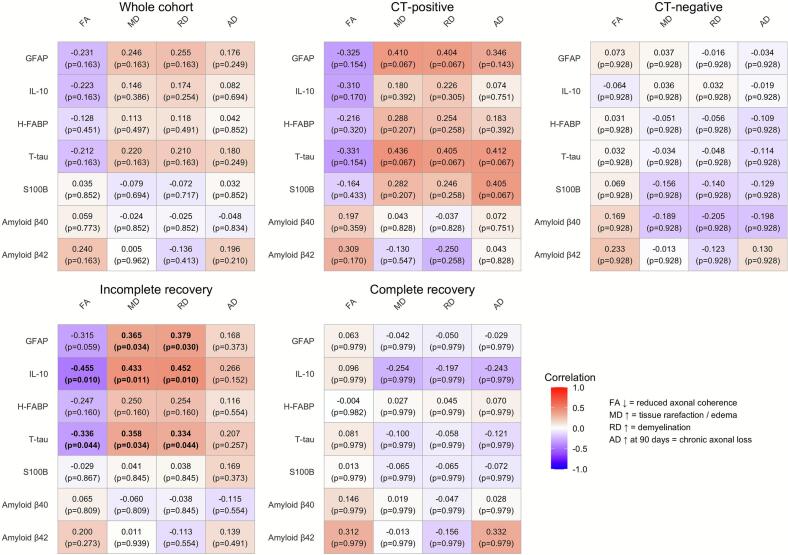
Correlation plot of biomarkers and DTI metrics in all patients, CT-positive cohort, CT-negative cohort, incomplete recovery cohort, complete recovery cohort. GFAP, glial fibrillary acidic protein; IL-10, interleukin 10; H-FABP, heart fatty-acid binding protein; T-tau, total tau; S100B, S100 calcium binding protein; Aβ42/Aβ40, amyloid beta 42/40; FA, fractional anisotropy; MD, mean diffusivity; RD, radial diffusivity; AD, axial diffusivity.

### 3.2 DTI metrics

The median levels of DTI metrics in subgroups are shown in Supplementary **table S2**. FA values were lower in CT-positive patients compared to CT-negative patients (p < 0.001), while MD and RD were higher in the CT-positive group (p = 0.007, p = 0.001, respectively), but no difference was observed in AD (Fig. 3). When the DTI metrics were compared between patients with incomplete recovery and complete recovery, no significant differences were observed (Fig. 3).

### 3.3 Correlations between biomarker levels and DTI metrics

Correlations of biomarker levels with FA, MD, RD and AD are shown in Fig. 4 and further in detail in Supplementary **table S3A-D**. Biomarkers GFAP, IL-10 and T-tau showed weak-moderate negative correlations with FA. Among these, correlations for IL-10 and T-tau were statistically significant even after correction for multiple comparisons in the incomplete recovery group, whereas the correlation of GFAP was slightly below significance threshold. Correlations were also seen in whole cohort and CT-positive patients, but they did not reach statistical significance after correcting for multiple comparisons. A weak positive correlation was found between the Aβ42 and FA levels in the whole cohort, although not significant after correction.

MD showed weak-moderate positive correlations with the admission levels of GFAP, IL-10 and T-tau in incomplete recovery group, with p-values reaching statistical significance after multiple correction. Similar associations were found for GFAP and T-Tau in CT-positive and whole cohort groups. In the incomplete recovery group, the strongest correlation was found for IL-10 and DTI metrics, but in the CT-positive and whole cohort group the correlations were not significant before multiple correction.

RD showed a similar pattern of positive correlation as MD, with the admission levels of GFAP, IL-10 and T-tau having weak-moderate positive correlations, only reaching statistical significance after correction in the incomplete recovery group.

AD had weak-moderate positive correlations with the admission levels of GFAP, T-tau and S100B only in the CT-positive group, and those correlations were not statistically significant after correction. Aβ42/Aβ40 ratio did not have significant correlations with DTI metrics in all mTBI patients or subgroups (Supplementary **table S4**).

No significant correlations were found between the levels of admission blood biomarkers and the DTI metrics in CT-negative patients or patients with complete recovery. A separate analysis was performed in patients with the PTA of 24 h or less (n = 50) and in the subgroups of CT-positive (n = 16), CT-negative (n = 34), incomplete recovery (n = 25) and complete recovery (n = 25). In this patient group with PTA < 24 h, only isolated associations were observed, which were not consistent across the data and did not survive correction for multiple comparisons **(**Supplementary **table S5A-D)**. FA showed a moderate positive correlation with Aβ42 in the complete recovery group. MD did not have significant correlations with any biomarker when categorized into subgroups. RD had a weak negative correlation with the admission levels of S100B in the whole cohort and a moderate negative correlation with Aβ40 in the CT-negative subgroup. AD showed a moderate positive correlation with IL-10 in the incomplete recovery group and a strong positive correlation with Aβ40 in the CT-positive group.

## 4 Discussion

This study examined the association between the acute plasma levels of seven protein biomarkers of different origins with the development of WM alterations, assessed using post-acute DTI metrics from DW-MRI, as well as their relationship with clinical outcome. DW-MRI has been shown to be a promising imaging modality for detecting DAI and reduced WM integrity in TBIs of different severities after injury. (Yin et al., 2019, Hashim et al., 2017)Acute phases of TBI include primary trauma and direct cell disruptions, edema, hypertrophy and proliferation. Cellular alterations, such as neuronal cell death and axon degeneration, develop within weeks from the injury and lead to reduced directionality in the brain, which is seen as increased global diffusivity measures and reduced anisotropy. (Benjamini et al., 2021) As expected, patients with CT-positive head scans on admission had a significantly lower FA and higher MD and RD in the post-acute phase of mTBI, as assessed by DW-MRI. This can reflect a more severe injury than in a typical patient with mTBI, since development of DAI is associated with injury severity. (Kumar et al., 2009) Earlier studies on mTBI assessing DTI values in specific fiber tracts have shown associations of FA, MD and RD with post-injury symptoms. (Kumar et al., 2009, Mohammadian et al., 2020, Roine et al., 2022) In this study, no differences were observed between the outcome groups in global DTI metrics, which could indicate that a global approach may not be specific enough to detect changes in regions relevant to outcome.

GFAP is a cytosolic protein found in astrocytes located in central nervous system. (Posti and Tenovuo, 2022) Elevated levels of GFAP have been reported to be negatively associated with FA and positively with MD, RD and AD in multiple WM tracts in subacute phase one month after injury. (Shahim et al., 2020) The results of the present study support previous findings that admission GFAP is associated with reduced WM integrity, but it remains unclear whether this reflects progressive DAI development or persistent changes following acute damage. These results were mostly driven by the CT-positive and incomplete recovery group. TBI results in a rapid release of GFAP into the blood and high levels can be found for a prolonged duration. (Janigro et al., 2022) Although GFAP is a known marker of astrogliosis, it remains unclear whether blood levels of GFAP accurately reflect astroglial activation in the brain. Persistently high GFAP levels may indicate ongoing brain injury or astrogliosis. (Shahim et al., 2020, Yang and Wang, 2015, Abdelhak et al., 2022, Graham, 2022) Studies have suggested that GFAP is also able to predict unfavorable (GOSE ≤ 4) recovery and return to work after mTBI, but it seems that it is not able to predict full recovery, as also noted in the present study. (Metting et al., 2012, Diaz-Arrastia et al., 2014).

The anti-inflammatory protein IL-10 is a cytokine expressed in the neurochemical inflammatory reaction following TBI. We found that it correlated with DTI metrics FA, MD, and RD, especially in the incomplete recovery group. This could represent the magnitude of initial neuroinflammatory reaction, since IL-10 is found to increase simultaneously with pro-inflammatory cytokines (Kumar et al., 2015), resulting in reduced WM integrity. Increased levels of IL-10 have been linked to poor outcome and mortality in severe TBI (sTBI) (Mahatma et al., 2012, Kumar et al., 2015), whereas its outcome prediction ability in mTBI has not been reported to be sufficient (Lagerstedt et al., 2020). In this study it was elevated in CT-positive patients, which is in line with previous studies. (Mahatma et al., 2012, Lagerstedt et al., 2018).

Tau is a microtubule-associated protein found in axon terminals of non-myelinated interneurons of the cortex. (Trojanowski et al., 1989) It is generally believed that DAI affects large, myelinated axons in the brain, but it is also suggested that smaller unmyelinated axons would be vulnerable to TBI. (Johnson et al., 2013, Sharp et al., 2014) T-tau appears to have a peak at 12–24 h after injury (Posti and Tenovuo, 2022) and a second peak at 36 h (Shahim et al., 2014). Similarly to earlier studies, T-tau showed a negative association with FA and positive associations with MD, RD, and AD at admission. (Wu et al., 2023, Kawata et al., 2020) We observed correlations between T-tau and DTI metrics in the CT-positive and incomplete recovery groups. Based on the earlier evidence, the associations seem to be significantly dependent on the time of injury to sampling. (Shahim et al., 2020) While we observed correlations between admission T-tau levels and post-acute DTI metrics, in another study using T-tau levels and imaging obtained at 30 days, no correlation was detected. (Shahim et al., 2020) T-tau has demonstrated outcome prediction ability especially in sTBI. (Liliang et al., 2010).

Amyloid precursor protein is a membrane protein in the brain that is forms β-amyloid peptides Aβ40 and Aβ42 as a part of normal metabolism. (Blennow et al., 2006) TBI can lead to imbalance in the β-amyloid metabolism and lead to accumulation of these proteins into swollen, disconnected axon terminals. (Johnson et al., 2013) In earlier studies of chronic mTBI, Aβ42 did not have any significant correlations but rather a trend of positive correlation with FA and negative correlations with MD, RD, and AD, which were mainly seen in moderate-severe injuries, and not in mild TBI. (Lippa et al., 2019) In the present study we found a weak positive correlation with admission Aβ42 and post-acute FA in whole cohort without reaching statistical significance after multiple correction. This may be an individual false-positive result of multiple analyses, since no correlation was seen in subgroup analyses. There were no significant correlations between Aβ42/Aβ40 ratio and DTI metrics. The levels of Aβ40 and Aβ42 increase in the plasma after TBI (Mondello et al., 2014), but studies have reported later peaks at 30 days indicating a disrupted amyloid clearance pathway in some patients. (Bogoslovsky et al., 2017) The initial levels at admission may not reflect the amyloid deposition and some studies also suggest that in mTBI pathology, amyloids are not a significant component. (Zetterberg et al., 2006) Amyloid-β markers are associated with outcome in sTBI (Franz et al., 2003), but no associations have been found with outcome in mTBI. (Shahim et al., 2016).

H-FABP is found in neurons and in the heart (Walder et al., 2013), and it is found to associate with head CT findings in mTBI patients. (Lagerstedt et al., 2017) No correlation with H-FABP and DTI metrics was found in this study. An earlier study showed limited value for H-FABP in outcome prediction. The study had partially same cohort as the current study but excluded those sampled after 24 h. (Lagerstedt et al., 2020).

S100B is released from astrocytes following TBI. (Marchi et al., 2003) A study investigating the admission levels of S100B and MRI abnormalities at 3-month follow-up did not find any relationship between axonal injuries and S100B (Metting et al., 2012), which is consistent with the present study. S100B has a rapid degradation and a half-life of 2 h – 6 h is suggested. (Thelin et al., 2017) In the present study, the median time elapsed from injury to sampling exceeded the half-life of S100B and the results may not capture the true peak values.

A major strength of this well-characterized, prospectively collected study is that multiple biomarkers from various cell types were used. As a limitation in this study, it should be noted that the classification of TBI severity was solely based on the worst GCS score recorded and the patient selection for the study cohort did not consider CT findings or PTA duration. The TBI severity classification has been debated for years, which has complicated the interpretation of TBI studies. (Tenovuo et al., 2021, Uchino et al., 2001) New characterization system suggests incorporating biomarkers and advanced imaging methods in the future to improve characterization of TBI. (Bazarian et al., 2025, Mac Donald et al., 2025, Whitehouse et al., 2025) During the recruitment for the current study, patients with the mildest TBIs were often discharged from the hospital before the enrolment was possible. We had more CT-positive patients than a typical mTBI cohort, and some patients even required surgery. As a result, this study represents mTBIs from the severe end and does not represent the typical patient with mTBI. To account for these factors, we divided the data into subgroups of CT-positive and CT-negative patients. The results seem to be driven by more severe cases since significant results were found only in the CT-positive and incomplete recovery groups.

The duration of PTA was over 24 h in some patients, which according to many classifications would mean at least moderate TBI. PTA was evaluated retrospectively at the follow-up visit, which is less reliable than a prospective assessment. We separately analyzed patients with PTA < 24 h and did not observe any correlations between biomarkers and the DTI metrics even when categorized into CT-positive and CT-negative subgroups. PTA has been found to be a strong outcome prediction in TBI (De Simoni et al., 2016), but not necessarily in mTBI with PTA < 24 h. (Ponsford et al., 2000, van der Naalt et al., 2017)Another limitation that this is a single-center study with only one time point for blood sampling and the small sample size, and more research is needed to validate these results in a larger cohort.

There were considerable variations in the delays between the time from injury to sampling. Since the kinetics for each biomarker are different, this may affect the levels, especially with biomarkers with rapid degradation, such as H-FABP, IL-10, and S100B. Another limitation is the presence of extracranial trauma in many patients, which can impact the levels of biomarkers with extracranial sources, for example, S100B. There was no difference between outcome groups with isolated TBIs or non-isolated, and the levels of ISS did not significantly differ between groups. It should be noted that the outcome assessment time varied between 6 to 12 months, and some patients could have recovered fully within a longer follow-up period. In this study, we used GOSE for functional outcome assessment, which might not be sensitive enough to capture all post-injury symptoms. Herein, other outcome measures need to be implemented in future research. Most patients recover quickly from their injury and studies suggest that most of the patients that are symptomatic at 6 months do not fully recover. (van Der Naalt et al., 1999).

## 5 Conclusions

In this study investigating seven blood-based biomarkers in TBI patients presenting with GCS of 13–15, the admission levels of IL-10 and T-tau showed weak-moderate negative correlations with post-acute FA, while GFAP, IL-10 and T-tau showed positive correlations with MD and RD in incompletely recovered patients. Similar trends were observed in the whole cohort and in the CT-positive cohort, although they did not reach statistical significance after correction for multiple comparisons. A significant correlation between the biomarker levels measured on admission and the DAI-related microstructural injury may suggest that also these biomarkers––of which GFAP and IL-10 are non-axonal––are associated with the development of DAI. If validated in future studies, these biomarkers may aid in identifying patients who could benefit from closer follow-up or advanced imaging such as DW-MRI. However, because their levels did not differ between patients with complete and incomplete recovery in this cohort, they may not be sufficient alone for clinical decision-making. Given the complex and dynamic nature of TBI, single blood samples obtained at variable times post-injury are probably inadequate to capture the full biological evolution underlying long-term outcomes.

**Conflict of interest**

KB has served as a consultant, at advisory boards, or at data monitoring committees for Abcam, Axon, BioArctic, Biogen, JOMDD/Shimadzu. Julius Clinical, Lilly, MagQu, Novartis, Ono Pharma, Pharmatrophix, Prothena, Roche Diagnostics, and Siemens Healthineers, and is a co-founder of Brain Biomarker Solutions in Gothenburg AB (BBS), which is a part of the GU Ventures Incubator Program, outside the work presented in this paper. VFJN has held an investigator lead grant with Roche Pharmaceuticals and received inkind support from Upfront Diagnostics.

HZ has served at scientific advisory boards and/or as a consultant for Abbvie, Acumen, Alector, Alzinova, ALZpath, Amylyx, Annexon, Apellis, Artery Therapeutics, AZTherapies, Cognito Therapeutics, CogRx, Denali, Eisai, LabCorp, Merry Life, Nervgen, Novo Nordisk, Optoceutics, Passage Bio, Pinteon Therapeutics, Prothena, Quanterix, Red Abbey Labs, reMYND, Roche, Samumed, Siemens Healthineers, Triplet Therapeutics, and Wave, has given lectures sponsored by Alzecure, BioArctic, Biogen, Cellectricon, Fujirebio, Lilly, Novo Nordisk, Roche, and WebMD, and is a co-founder of Brain Biomarker Solutions in Gothenburg AB (BBS), which is a part of the GU Ventures Incubator Program (outside submitted work). OT has served as a consultant for NeurotraumaSciences and Abbott. JPP has received speaker’s fees from Sanofi S.A., the Finnish Medical Association, Wellbeing services county of North Karelia, and Finnish Association of Otorhinolaryngology – Head and Neck Surgery and travel expenses reimbursement and expert fee from the National Institute of Neurological Disorders and Stroke. The views expressed in this manuscript are solely those of the authors and do not necessarily represent the official positions of their respective institutions.

## CRediT authorship contribution statement

**Malla Mononen:** Writing – original draft, Investigation, Formal analysis. **Mehrbod Mohammadian:** Writing – review & editing, Formal analysis. **Iftakher Hossain:** Writing – review & editing. **Timo Roine:** Writing – review & editing, Formal analysis. **Olli Tenovuo:** Writing – review & editing, Resources, Data curation, Conceptualization. **Kaj Blennow:** Writing – review & editing, Resources. **Jessica Gill:** Writing – review & editing, Resources. **Mark van Gils:** Writing – review & editing, Resources, Data curation. **Peter Hutchinson:** Writing – review & editing, Resources. **Teemu M. Luoto:** Writing – review & editing, Data curation. **Henna-Riikka Maanpää:** Writing – review & editing. **David K. Menon:** Writing – review & editing, Resources. **Virginia F.J. Newcombe:** Writing – review & editing, Resources. **Rahul Raj:** Writing – review & editing, Data curation. **Jean-Charles Sanchez:** Writing – review & editing, Resources. **Riikka S.K. Takala:** Writing – review & editing, Resources, Data curation. **Jussi Tallus:** Writing – review & editing, Resources, Data curation. **Henrik Zetterberg:** Writing – review & editing, Resources. **Jussi P. Posti:** Writing – review & editing, Supervision, Resources, Data curation, Conceptualization.

## Declaration of competing interest

The authors declare that they have no known competing financial interests or personal relationships that could have appeared to influence the work reported in this paper.

## Data Availability

Data will be made available on request.
