# Supplementary material for: Association of acute blood biomarkers with diffusion tensor imaging and outcome in patients with traumatic brain injury presenting with GCS of 13–15
Source: Neuroimage Clin. 2025 Dec 18;49:103934. doi: 10.1016/j.nicl.2025.103934 (PMC12811550; doi:10.1016/j.nicl.2025.103934)
Supplement: Supplementary Data 1 [file mmc1.docx]

| **Supplementary table 1.** The levels of acute biomarkers in all mTBI patients and mTBI subgroups | | | | | | | |
| --- | --- | --- | --- | --- | --- | --- | --- |
|  | GFAP | IL-10 | H-FABP | T-tau | S100B | Aβ42 | Aβ40 |
| Group | median [IQR] | median [IQR] | median [IQR] | median [IQR] | median [IQR] | median [IQR] | median [IQR] |
| All patients | 1054.0 [229.0*–*2672.0] | 0.5 [0.2*–*1.4] | 5.5 [3.7*–*15.7] | 2.6 [1.3*–*6.2] | 78.1 [45.1*–*120.5] | 16.7 [11.8*–*22.2] | 17.3 [12.7*–*24.5] |
| CT-negative | 472.0 [131.1*–*1396.6] | 0.4 [0.2*–*0.8] | 4.4 [2.9–10] | 2.1 [1.1*–*3.1] | 80.6 [52.6*–*119.4] | 16.8 [13.1*–*18.9] | 16.7 [12.2*–*20.4] |
| CT-positive | 2727.9 [876.9–11221.3] | 0.7 [0.3–2.3] | 6.6 [4.8–18.5] | 4.0 [1.9–11.3] | 73.0 [40.6–119.3] | 16.4 [10.2– 24.0] | 19.6 [13.3– 27.5] |
| Compete recovery | 612.1 [231.1–2226.8] | 0.4 [0.3–1.4] | 5.9 [3.4–13.5] | 2.7 [1.5–4.3] | 77.6 [52.4–116.5] | 17.2 [12.5–23.8] | 18.2 [13.3–25.2] |
| Incomplete recovery | 1396.6 [233.7–5240.7] | 0.5 [0.2–1.3] | 5.5 [3.7–16.1] | 2.5 [1.3–8.6] | 78.5 [43.5–121.8] | 15.8 [11.8–20.4] | 17.1 [12.4–24.4] |

mTBI, mild traumatic brain injury; CT, computed tomography; IQR, interquartile range; GFAP, glial fibrillary acidic protein; IL-10, interleukin 10; H-FABP, heart fatty-acid binding protein; T-Tau, total tau; S100B, S100 calcium binding protein; Aβ42, amyloid β42; Aβ40, amyloid β40.

| **Supplementary table 2.** The levels of DTI metrics in all mTBI patients and mTBI subgroups | | | | |
| --- | --- | --- | --- | --- |
|  | FA | MD (x10^-3^ mm^2^/s) | RD (x10^-3^ mm^2^/s) | AD (x10^-3^ mm^2^/s) |
| Group | median [IQR] | median [IQR] | median [IQR] | median [IQR] |
| All patients | 0.472 [0.442 - 0.490] | 0.726 [0.712 - 0.743] | 0.523 [0.504 - 0.551] | 1.126 [1.106 - 1.150] |
| CT-negative | 0.481 [0.462 - 0.495] | 0.723 [0.710 - 0.733] | 0.515 [0.502 - 0.534] | 1.124 [1.098 - 1.143] |
| CT-positive | 0.463 [0.431 - 0.477] | 0.734 [0.715 - 0.772] | 0.536 [0.515 - 0.582] | 1.126 [1.109 - 1.153] |
| Complete recovery | 0.470 [0.458 - 0.495] | 0.729 [0.712 - 0.741] | 0.529 [0.499- 0.545] | 1.125 [1.092 - 1.152] |
| Incomplete recovery | 0.472 [0.442 - 0.486] | 0.724 [0.712 - 0.743] | 0.523 [0.504 - 0.566] | 1.126 [1.110 - 1.149] |

mTBI, mild traumatic brain injury; DTI, diffusion tensor imaging; CT, computed tomography; FA, fractional anisotropy; MD, mean diffusivity; RD, radial diffusivity; AD, axial diffusivity; SD, standard deviation.

| **Supplementary table 3A**. Correlation between fractional anisotropy (FA) and biomarkers in all mTBI patients and mTBI subgroups | | | | | | | | | | |
| --- | --- | --- | --- | --- | --- | --- | --- | --- | --- | --- |
|  | All patients | | CT-positive | | CT-negative | | Incomplete recovery | | Complete recovery | |
| **Biomarker** | **Spearman ρ** | **p-value** | **Spearman ρ** | **p-value** | **Spearman ρ** | **p-value** | **Spearman ρ** | **p-value** | **Spearman ρ** | **p-value** |
| GFAP | -0.231 | 0.163 | -0.325 | 0.154 | 0.073 | 0.929 | -0.315 | 0.059 | 0.063 | 0.979 |
| IL-10 | -0.223 | 0.163 | -0.310 | 0.170 | -0.064 | 0.929 | **-0.455** | **0.010** | 0.096 | 0.979 |
| H-FABP | -0.128 | 0.451 | -0.216 | 0.320 | 0.031 | 0.929 | -0.247 | 0.160 | -0.004 | 0.982 |
| T-tau | -0.212 | 0.163 | -0.331 | 0.067 | 0.032 | 0.929 | **-0.336** | **0.044** | 0.081 | 0.979 |
| S100B | 0.035 | 0.852 | -0.164 | 0.433 | 0.069 | 0.929 | -0.029 | 0.867 | 0.013 | 0.979 |
| Aβ42 | 0.240 | 0.163 | 0.309 | 0.170 | 0.233 | 0.929 | 0.200 | 0.273 | 0.312 | 0.979 |
| Aβ40 | 0.059 | 0.773 | 0.197 | 0.359 | 0.169 | 0.929 | 0.065 | 0.809 | 0.146 | 0.979 |

| **Supplementary table 3B**. Correlation between mean diffusivity (MD) and biomarkers in all mTBI patients and mTBI subgroups | | | | | | | | | | |
| --- | --- | --- | --- | --- | --- | --- | --- | --- | --- | --- |
|  | All patients | | CT-positive | | CT-negative | | Incomplete recovery | | Complete recovery | |
| **Biomarker** | **Spearman ρ** | **p-value** | **Spearman ρ** | **p-value** | **Spearman ρ** | **p-value** | **Spearman ρ** | **p-value** | **Spearman ρ** | **p-value** |
| GFAP | 0.246 | 0.163 | 0.410 | 0.067 | 0.037 | 0.928 | **0.365** | **0.034** | -0.042 | 0.979 |
| IL-10 | 0.146 | 0.385 | 0.180 | 0.392 | 0.036 | 0.928 | **0.433** | **0.011** | -0.254 | 0.979 |
| H-FABP | 0.113 | 0.497 | 0.288 | 0.207 | -0.051 | 0.928 | 0.250 | 0.160 | 0.027 | 0.979 |
| T-tau | 0.220 | 0.163 | 0.436 | 0.067 | -0.034 | 0.928 | **0.358** | **0.039** | -0.100 | 0.979 |
| S100B | -0.079 | 0.694 | 0.282 | 0.207 | -0.156 | 0.928 | 0.041 | 0.845 | -0.065 | 0.979 |
| Aβ42 | 0.005 | 0.961 | -0.130 | 0.547 | -0.013 | 0.928 | 0.011 | 0.939 | -0.013 | 0.979 |
| Aβ40 | -0.024 | 0.852 | 0.043 | 0.828 | -0.189 | 0.928 | -0.060 | 0.809 | 0.019 | 0.979 |

| **Supplementary table 3C**. Correlation between radial diffusivity (RD) and biomarkers in all mTBI patients and mTBI subgroups | | | | | | | | | | |
| --- | --- | --- | --- | --- | --- | --- | --- | --- | --- | --- |
|  | All patients | | CT-positive | | CT-negative | | Incomplete recovery | | Complete recovery | |
| **Biomarker** | **Spearman ρ** | **p-value** | **Spearman ρ** | **p-value** | **Spearman ρ** | **p-value** | **Spearman ρ** | **p-value** | **Spearman ρ** | **p-value** |
| GFAP | 0.255 | 0.163 | 0.404 | 0.067 | -0.016 | 0.928 | **0.379** | **0.030** | -0.050 | 0.979 |
| IL-10 | 0.174 | 0.254 | 0.226 | 0.305 | 0.032 | 0.928 | **0.452** | **0.010** | -0.197 | 0.979 |
| H-FABP | 0.118 | 0.491 | 0.254 | 0.258 | -0.056 | 0.928 | 0.254 | 0.160 | 0.045 | 0.979 |
| T-tau | 0.210 | 0.163 | 0.405 | 0.067 | -0.048 | 0.928 | **0.334** | **0.044** | -0.058 | 0.979 |
| S100B | -0.072 | 0.717 | 0.246 | 0.258 | -0.140 | 0.928 | 0.038 | 0.845 | -0.065 | 0.979 |
| Aβ42 | -0.136 | 0.413 | -0.250 | 0.258 | -0.123 | 0.928 | -0.113 | 0.554 | -0.156 | 0.979 |
| Aβ40 | -0.025 | 0.852 | -0.037 | 0.828 | -0.205 | 0.928 | -0.038 | 0.845 | -0.047 | 0.979 |

| **Supplementary table 3D**. Correlation between axial diffusivity (AD) and biomarkers in all mTBI patients and mTBI subgroups | | | | | | | | | | |
| --- | --- | --- | --- | --- | --- | --- | --- | --- | --- | --- |
|  | All patients | | CT-positive | | CT-negative | | Incomplete recovery | | Complete recovery | |
| **Biomarker** | **Spearman ρ** | **p-value** | **Spearman ρ** | **p-value** | **Spearman ρ** | **p-value** | **Spearman ρ** | **p-value** | **Spearman ρ** | **p-value** |
| GFAP | 0.176 | 0.249 | 0.346 | 0.143 | -0.034 | 0.928 | 0.168 | 0.373 | -0.029 | 0.979 |
| IL-10 | 0.082 | 0.694 | 0.074 | 0.751 | -0.019 | 0.928 | 0.266 | 0.152 | -0.243 | 0.979 |
| H-FABP | 0.042 | 0.852 | 0.183 | 0.392 | -0.109 | 0.928 | 0.116 | 0.554 | 0.070 | 0.979 |
| T-tau | 0.180 | 0.249 | 0.412 | 0.067 | -0.114 | 0.928 | 0.207 | 0.257 | -0.121 | 0.979 |
| S100B | 0.032 | 0.852 | 0.405 | 0.067 | -0.129 | 0.928 | 0.169 | 0.373 | -0.072 | 0.979 |
| Aβ42 | 0.196 | 0.210 | 0.043 | 0.828 | 0.130 | 0.928 | 0.139 | 0.491 | 0.332 | 0.979 |
| Aβ40 | -0.048 | 0.834 | 0.072 | 0.751 | -0.198 | 0.928 | -0.115 | 0.554 | 0.028 | 0.979 |

Statistically significant in bold. mTBI, mild traumatic brain injury; CT, computed tomography; GFAP, glial fibrillary acidic protein; IL-10, interleukin 10; H-FABP, heart fatty-acid binding protein; T-Tau, total tau; S100B, S100 calcium binding protein; Aβ42, Amyloid β42; Aβ40, Amyloid β40.

| **Supplementary table 4**. Correlation between Aβ42/Aβ40 ratio and DTI metrics all mTBI patients and mTBI subgroups | | | | | | | | | | |
| --- | --- | --- | --- | --- | --- | --- | --- | --- | --- | --- |
|  | All patients | | CT-positive | | CT-negative | | Incomplete recovery | | Complete recovery | |
| **DTI metric** | **Spearman ρ** | **p-value** | **Spearman ρ** | **p-value** | **Spearman ρ** | **p-value** | **Spearman ρ** | **p-value** | **Spearman ρ** | **p-value** |
| FA | 0.108 | 0.536 | 0.097 | 0.708 | -0.016 | 0.928 | 0.071 | 0.815 | 0.101 | 0.996 |
| MD | 0.043 | 0.833 | -0.130 | 0.600 | 0.123 | 0.928 | 0.066 | 0.815 | 0.001 | 0.996 |
| RD | -0.053 | 0.833 | -0.153 | 0.517 | 0.077 | 0.928 | -0.033 | 0.863 | -0.048 | 0.996 |
| AD | 0.181 | 0.261 | 0.015 | 0.931 | 0.204 | 0.928 | 0.194 | 0.319 | 0.215 | 0.996 |

Statistically significant in bold. Aβ42, amyloid β42; Aβ40, amyloid β40; mTBI, mild traumatic brain injury; CT, computed tomography; FA, fractional anisotropy; MD, mean diffusivity; RD, radial diffusivity; AD, axial diffusivity.

| **Supplementary table 5A**. Correlation between biomarker levels and FA in patients with mTBI and a duration of less than 24 hours of posttraumatic amnesia | | | | | | | | | | |
| --- | --- | --- | --- | --- | --- | --- | --- | --- | --- | --- |
|  | All patients | | CT-positive | | CT-negative | | Incomplete recovery | | Complete recovery | |
| **Biomarker** | **Spearman ρ** | **p-value** | **Spearman ρ** | **p-value** | **Spearman ρ** | **p-value** | **Spearman ρ** | **p-value** | **Spearman ρ** | **p-value** |
| GFAP | -0.008 | 0.967 | 0.018 | 0.950 | 0.259 | 0.514 | 0.129 | 0.822 | 0.072 | 0.887 |
| IL-10 | 0.064 | 0.967 | 0.174 | 0.910 | 0.086 | 0.777 | -0.145 | 0.822 | 0.167 | 0.843 |
| H-FABP | 0.037 | 0.967 | -0.276 | 0.853 | 0.198 | 0.513 | -0.017 | 0.939 | 0.013 | 0.956 |
| T-tau | 0.073 | 0.967 | 0.298 | 0.853 | 0.173 | 0.535 | 0.130 | 0.822 | 0.035 | 0.943 |
| S100B | 0.232 | 0.813 | 0.308 | 0.853 | 0.161 | 0.556 | 0.190 | 0.822 | 0.098 | 0.887 |
| Aβ42 | 0.269 | 0.657 | 0.529 | 0.733 | 0.309 | 0.514 | 0.244 | 0.822 | 0.427 | 0.843 |
| Aβ40 | -0.013 | 0.967 | 0.074 | 0.923 | 0.280 | 0.514 | -0.117 | 0.831 | 0.199 | 0.843 |

| **Supplementary table 5B**. Correlation between biomarker levels and MD in patients with mTBI and a duration of less than 24 hours of posttraumatic amnesia | | | | | | | | | | |
| --- | --- | --- | --- | --- | --- | --- | --- | --- | --- | --- |
|  | All patients | | CT-positive | | CT-negative | | Incomplete recovery | | Complete recovery | |
| **Biomarker** | **Spearman ρ** | **p-value** | **Spearman ρ** | **p-value** | **Spearman ρ** | **p-value** | **Spearman ρ** | **p-value** | **Spearman ρ** | **p-value** |
| GFAP | 0.056 | 0.967 | -0.080 | 0.923 | -0.052 | 0.873 | 0.075 | 0.891 | -0.205 | 0.843 |
| IL-10 | -0.032 | 0.967 | -0.134 | 0.910 | -0.092 | 0.777 | 0.325 | 0.822 | -0.354 | 0.843 |
| H-FABP | -0.008 | 0.967 | 0.465 | 0.766 | -0.196 | 0.514 | 0.161 | 0.822 | 0.017 | 0.956 |
| T-tau | -0.151 | 0.967 | -0.234 | 0.853 | -0.221 | 0.514 | -0.268 | 0.822 | -0.246 | 0.843 |
| S100B | -0.266 | 0.657 | 0.134 | 0.910 | -0.253 | 0.514 | -0.339 | 0.822 | -0.195 | 0.843 |
| Aβ42 | 0.033 | 0.967 | -0.112 | 0.910 | 0.031 | 0.934 | 0.177 | 0.822 | -0.090 | 0.887 |
| Aβ40 | 0.045 | 0.967 | 0.485 | 0.732 | -0.347 | 0.514 | 0.091 | 0.865 | -0.036 | 0.943 |

| **Supplementary table 5C**. Correlation between biomarker levels and RD in patients with mTBI and a duration of less than 24 hours of posttraumatic amnesia | | | | | | | | | | |
| --- | --- | --- | --- | --- | --- | --- | --- | --- | --- | --- |
|  | All patients | | CT-positive | | CT-negative | | Incomplete recovery | | Complete recovery | |
| **Biomarker** | **Spearman ρ** | **p-value** | **Spearman ρ** | **p-value** | **Spearman ρ** | **p-value** | **Spearman ρ** | **p-value** | **Spearman ρ** | **p-value** |
| GFAP | 0.018 | 0.967 | -0.041 | 0.923 | -0.205 | 0.514 | -0.018 | 0.939 | -0.175 | 0.843 |
| IL-10 | -0.086 | 0.967 | -0.172 | 0.910 | -0.136 | 0.643 | 0.246 | 0.822 | -0.275 | 0.843 |
| H-FABP | -0.035 | 0.967 | 0.418 | 0.853 | -0.210 | 0.514 | 0.062 | 0.907 | 0.078 | 0.887 |
| T-tau | -0.113 | 0.967 | -0.229 | 0.853 | -0.214 | 0.514 | -0.276 | 0.822 | -0.105 | 0.887 |
| S100B | -0.293 | 0.657 | -0.043 | 0.923 | -0.256 | 0.514 | -0.301 | 0.822 | -0.212 | 0.843 |
| Aβ42 | -0.099 | 0.967 | -0.240 | 0.853 | -0.096 | 0.777 | 0.028 | 0.939 | -0.258 | 0.843 |
| Aβ40 | 0.006 | 0.967 | 0.258 | 0.853 | -0.413 | 0.514 | 0.100 | 0.865 | -0.144 | 0.843 |

| **Supplementary table 5D**. Correlation between biomarker levels and AD in patients with mTBI and a duration of less than 24 hours of posttraumatic amnesia | | | | | | | | | | |
| --- | --- | --- | --- | --- | --- | --- | --- | --- | --- | --- |
|  | All patients | | CT-positive | | CT-negative | | Incomplete recovery | | Complete recovery | |
| **Biomarker** | **Spearman ρ** | **p-value** | **Spearman ρ** | **p-value** | **Spearman ρ** | **p-value** | **Spearman ρ** | **p-value** | **Spearman ρ** | **p-value** |
| GFAP | 0.130 | 0.967 | 0.052 | 0.923 | -0.013 | 0.945 | 0.159 | 0.822 | -0.153 | 0.843 |
| IL-10 | 0.134 | 0.967 | 0.226 | 0.853 | 0.022 | 0.938 | 0.432 | 0.822 | -0.181 | 0.843 |
| H-FABP | 0.102 | 0.967 | 0.313 | 0.853 | -0.056 | 0.873 | 0.215 | 0.822 | 0.197 | 0.843 |
| T-tau | -0.092 | 0.967 | -0.140 | 0.910 | -0.175 | 0.535 | -0.212 | 0.822 | -0.204 | 0.843 |
| S100B | -0.099 | 0.967 | 0.358 | 0.853 | -0.192 | 0.514 | -0.246 | 0.822 | 0.069 | 0.887 |
| Aβ42 | 0.190 | 0.967 | 0.123 | 0.910 | 0.197 | 0.514 | 0.161 | 0.822 | 0.283 | 0.843 |
| Aβ40 | 0.088 | 0.967 | 0.740 | 0.070 | -0.303 | 0.514 | 0.040 | 0.939 | 0.084 | 0.887 |

Statistically significant in bold. mTBI, mild traumatic brain injury; CT, computed tomography; FA, fractional anisotropy; MD, mean diffusivity; RD, radial diffusivity; AD, axial diffusivity; GFAP, glial fibrillary acidic protein; IL-10, interleukin 10; H-FABP, heart fatty-acid binding protein; T-Tau, total tau; S100B, S100 calcium binding protein; Aβ42, amyloid β42; Aβ40, amyloid β40.
